# Identification of Dendrobium Using Laser-Induced Breakdown Spectroscopy in Combination with a Multivariate Algorithm Model

**DOI:** 10.3390/foods13111676

**Published:** 2024-05-27

**Authors:** Tingsong Zhang, Ziyuan Liu, Qing Ma, Dong Hu, Yujia Dai, Xinfeng Zhang, Zhu Zhou

**Affiliations:** 1 College of Opto-Electro-Mechanical Engineering, Zhejiang A&F University, Hangzhou 311300, Chinaliuziyuan@zafu.edu.cn (Z.L.); daiyujia@zafu.edu.cn (Y.D.); 2 State Key Laboratory of Subtropical Silviculture, Zhejiang A&F University, Hangzhou 311300, China

**Keywords:** dendrobium, LIBS, feature selection, machine learning, t-SNE, classification

## Abstract

Dendrobium, a highly effective traditional Chinese medicinal herb, exhibits significant variations in efficacy and price among different varieties. Therefore, achieving an efficient classification of Dendrobium is crucial. However, most of the existing identification methods for Dendrobium make it difficult to simultaneously achieve both non-destructiveness and high efficiency, making it challenging to truly meet the needs of industrial production. In this study, we combined Laser-Induced Breakdown Spectroscopy (LIBS) with multivariate models to classify 10 varieties of Dendrobium. LIBS spectral data for each Dendrobium variety were collected from three circular medicinal blocks. During the data analysis phase, multivariate models to classify different Dendrobium varieties first preprocess the LIBS spectral data using Gaussian filtering and stacked correlation coefficient feature selection. Subsequently, the constructed fusion model is utilized for classification. The results demonstrate that the classification accuracy of 10 Dendrobium varieties reached 100%. Compared to Support Vector Machine (SVM), Random Forest (RF), and K-Nearest Neighbors (KNN), our method improved classification accuracy by 14%, 20%, and 20%, respectively. Additionally, it outperforms three models (SVM, RF, and KNN) with added Principal Component Analysis (PCA) by 10%, 10%, and 17%. This fully validates the excellent performance of our classification method. Finally, visualization analysis of the entire research process based on t-distributed Stochastic Neighbor Embedding (t-SNE) technology further enhances the interpretability of the model. This study, by combining LIBS and machine learning technologies, achieves efficient classification of Dendrobium, providing a feasible solution for the identification of Dendrobium and even traditional Chinese medicinal herbs.

## 1. Introduction

Dendrobium is acclaimed as the “soft gold” among traditional Chinese medicinal materials. In ancient times, “Shen Nong’s Herbal Classic” pointed out that Dendrobium has the effects of nourishing fluids, relieving thirst, alleviating pain, and eliminating edema. “Compendium of Materia Medica” also emphasized its efficacy in “strengthening yin, benefiting essence, long-term use thickens the intestines and stomach, lightens the body, and prolongs life.” In recent years, experts in the field of modern medicine have confirmed that Dendrobium possesses effects such as anti-cancer [1], anti-aging [2], enhancing immunity [3], and lowering blood sugar [4]. However, there are numerous varieties of Dendrobium, and the significant differences in the types and quantities of chemical components among different varieties directly lead to variations in pharmacological effects. Furthermore, it is challenging to identify the quality of Chinese medicinal materials based on the form and aroma of finished Chinese medicinal products. Many illegal entities exploit this loophole in the market by selling inferior medicinal materials as high-quality ones, profiting immensely. This phenomenon greatly disrupts the order of the supply chain of traditional Chinese medicine. However, the current classification of Dendrobium on the market mainly relies on manual methods. Such classification methods are highly subjective and slow. Researchers have begun further research on the classification of Dendrobium. They utilize technologies such as electronic nose (E-nose) [5,6], near-infrared spectroscopy (NIR) [7,8], nuclear magnetic resonance (NMR) [9], ultraviolet–visible spectroscopy [8,10], etc., to successfully classify multiple categories of Dendrobium. However, these technologies often suffer from deficiencies such as a lack of real-time capability, slow detection speed, long sample preparation time, sample damage, and high cost. Therefore, establishing a precise, efficient, and non-destructive classification method for Dendrobium species is of significant practical importance in the current traditional Chinese medicine industry.

The Laser-Induced Breakdown Spectroscopy (LIBS) technique effectively addresses the challenges that traditional detection methods mentioned above cannot overcome. LIBS generates high-temperature plasma by applying laser pulses to the tested sample [11,12]. The radiation spectrum emitted by plasma contains information on the composition and content of the substance in the tested sample. By analyzing these spectral data, it is possible to achieve a qualitative analysis of the tested sample. LIBS technology offers advantages such as high efficiency, in situ, and non-destructive. It is applied in various fields, including metal detection [13,14], scientific archaeology [15], environmental monitoring [16,17], and medical diagnostics [18]. Due to the variations in varieties and medicinal values of Chinese medicinal herbs, which can be attributed to differences in elemental compositions [19,20,21]. LIBS, with its capability of comprehensive elemental detection [22,23,24], can classify Chinese medicinal herbs by analyzing the elemental spatial information contained in the collected LIBS spectra. In recent years, several researchers have conducted relevant studies [25,26,27,28,29,30]. They have utilized LIBS technology for the detection of Chinese medicinal materials such as Dendrobium. Fang et al. [25] proposed a method for analyzing LIBS data of damaged medicinal leaves, achieving the quantification of Cd and Pb in them. Zhu et al. [26] used standard addition and internal standard methods to analyze LIBS spectral data, detecting harmful trace elements in licorice. However, analyzing LIBS data is a very complex task, and without strong professional knowledge, it is easy to cause analysis errors [27]. However, with the rapid rise of machine learning technology, its efficient analytical and learning capabilities have provided effective solutions to the aforementioned challenges. Liang et al. [28] used Random Forest (RF) to analyze LIBS and Infrared Spectroscopy (IR) data of Salvia miltiorrhiza to achieve classification tasks. Peng et al. [29] used residual neural networks to analyze the LIBS spectral data of white peonies to achieve rapid identification of the origin of white peonies. Zhang et al. [30] analyzed LIBS spectral data of Ginkgo biloba leaves using various machine learning models to achieve the origin traceability of Ginkgo biloba. These researchers utilized various machine learning methods for qualitative or quantitative analysis of LIBS spectral data from different substances. However, there are still challenges in using machine learning to analyze LIBS data for qualitative tasks.

Firstly, the main challenge limiting the accuracy of LIBS spectral data analysis has been the noise generated during the data collection process and the excessively high data dimensions. The factors contributing to the generation of noise in LIBS spectral data are mostly uncontrollable in practical experiments, such as fluctuations in laser amplitude and frequency, variations in external light sources and atmospheric conditions, and splattering of samples due to prolonged laser exposure [31,32]. Furthermore, a complete set of LIBS spectral data is often composed of over ten thousand spectral intensity values, leading to their high-dimensional complexity. However, for such high-dimensional and complex LIBS spectral data, the factors determining the analysis results often represent only a small portion of the entire dataset. Simultaneously, in terms of machine learning analysis, directly analyzing LIBS spectral data that include over ten thousand spectral intensity values and contain noise often encounters issues such as the “curse of dimensionality” and overfitting, making it challenging to accurately capture complex relationships within the data and affecting the analytical performance of the model [33,34]. Whether it is for LIBS technology or machine learning, reasonable and effective denoising and dimensionality reduction methods are crucial. However, in the past, researchers have mostly relied on manual spectral line selection in the analysis of LIBS spectral data to achieve dimensionality reduction and denoising effects. The manual selection process is often accompanied by drawbacks such as high theoretical and technical requirements, lengthy processing times, and subjectivity. Therefore, the handling of high-dimensional complexity is a crucial issue that urgently needs to be addressed in the analysis of LIBS spectral data.

Secondly, the mapping rules of a single machine learning model are no longer sufficient to support the analysis of such high-dimensional and complex data. Although the current analysis of LIBS spectral data by a single machine learning model has achieved certain effectiveness, there is still a long way to go in achieving the high precision standards required for real-world production. For real-world production, high precision implies high production efficiency and low-cost consumption. Therefore, the improvement of accuracy is of utmost importance in analysis tasks. In addition, how to achieve an increase in model analysis accuracy without sacrificing the lightweight attributes of the model is also a significant concern in the industry. This is because often, a more accurate model corresponds to a more complex model architecture, and a more complex model architecture implies the need for higher hardware investment and unavoidably lower analysis speed. Therefore, breaking free from the constraints of a single mapping rule and establishing a model that combines high precision with lightweight attributes is a pressing industrial challenge at present.

Finally, interpretability is a highly valued attribute in the analysis tasks of machine learning for researchers at present. It refers to the degree to which humans can understand the causal relationship between the input and output of a machine learning model [35,36]. An interpretable model allows stakeholders, including data researchers and non-experts, to understand the logic behind the model’s decisions. This understanding builds trust in machine learning among people and helps validate the reliability of the model. Therefore, achieving and enhancing the interpretability of models has always been a pursuit in the industry during the machine learning analysis process.

This paper combines LIBS technology with machine learning methods to achieve classification research on Dendrobium. Solutions are proposed to address three challenges: the high-dimensional complexity of LIBS spectral data, limitations associated with a singular machine learning mapping rule, and the realization and enhancement of model interpretability. Firstly, to address the noise and redundant information in LIBS data, along with the “curse of dimensionality” and overfitting challenges faced by machine learning, this study employs Gaussian filtering for the initial processing of underlying high-frequency noise. It then introduces the maximum mutual information correlation coefficient for spectral line selection in LIBS spectral data, enhancing selection efficiency while maintaining effective spectral lines. Additionally, the Spearman correlation coefficient is introduced on the selected results to eliminate redundant information between features, further achieving denoising and maximizing redundancy removal. Experimental results demonstrate that this method not only further improves analysis accuracy but also efficiently extracts LIBS spectral line information while effectively avoiding the risks of the “curse of dimensionality” and overfitting that machine learning may face. Secondly, to ensure the high precision and lightweight characteristics of the model to meet practical production requirements, this study establishes a simple yet effective fused classification model. It combines multiple lightweight classification models in parallel and provides feedback to the fused model based on the training results of the sub-models. The final fused model autonomously adjusts parameters based on feedback information from the sub-models. Research proves that the aforementioned fused model not only ensures model lightweight characteristics but also further enhances the analysis accuracy of LIBS spectral data. Eventually, we use t-distributed Stochastic Neighbor Embedding (t-SNE) [37,38] to visually and clearly analyze the entire experimental process. Through this step, the effectiveness and excellence of the aforementioned operations in the classification task are visually demonstrated, enhancing the interpretability of the model.

## 2. Experimental Setup and Methods

### 2.1. Preparation of Experimental Samples

We conducted a study using 10 varieties of Dendrobium cultivated at the State Key Laboratory of Subtropical Silviculture, Zhejiang A&F University. The identification codes for these ten Dendrobium varieties are D5, S1, S3, S6, S9, S13, S15, S23, 17*136 and 18*22 (* denotes hybridization of two varieties). For ease of understanding and differentiation, the subsequent text will uniformly abbreviate the identification codes of the aforementioned Dendrobium varieties as A, B, C, D, E, F, G, H, I, and J. Each of the ten varieties is treated as distinct, with the LIBS spectral data collected from pure samples of each Dendrobium variety rather than from mixed samples. Furthermore, morphological differences in Dendrobium samples may also contribute to errors during LIBS spectral acquisition. In consideration of the aforementioned factors, this study adopts the approach of grinding Dendrobium into powder and then compressing it into cakes for sample preparation. This method not only aligns with practical considerations but also effectively mitigates experimental errors arising from differences in sample morphology.

Firstly, we randomly divided ten independent samples of each Dendrobium variety into training, testing, and validation sets at proportions of 60%, 20%, and 20%, respectively. Specifically, the training set for each variety consisted of six independent Dendrobium samples, while the testing and validation sets for each variety each comprised two independent Dendrobium samples. Subsequently, all Dendrobium samples in each set were ground into powder and sieved through a 60-mesh sieve, followed by thorough mixing. The powdered samples were then placed on weighing paper, and 2 g of each sample were weighed using an electronic balance. The samples were subsequently compressed into circular discs with a diameter of 20 mm and a thickness of 2 mm using a tablet press under a pressure of 25 MPa for 30 min. This process was repeated to produce three circular medicinal blocks (Φ20 × 2 mm) weighing 2 g each for each Dendrobium variety. By uniformly preparing Dendrobium samples into powder cakes, this approach achieves not only the uniformity of sample morphology but also reduces noise errors during LIBS spectral acquisition caused by surface roughness.

### 2.2. Experimental Setup and Spectral Acquisition

**Figure 1:**
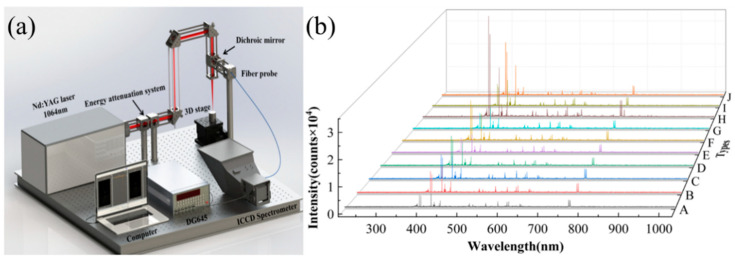
Schematic diagram of the experimental setup and sample spectra. (**a**) Experimental setup diagram; (**b**) LIBS spectra for 10 Dendrobium varieties.

The LIBS experimental setup is illustrated in Figure 1a. The setup consists of various components, including a nanosecond laser system (Libra, Coherent, Saxonburg, PA, USA), energy attenuation system, spectrometer (Mechelle 5000, Andor, UK), pulse delay trigger, 3D translation stage, optical lenses, and a computer. The femtosecond laser operates at a wavelength of 800 nm, with a pulse width of 50 fs, a repetition rate of 1.0 kHz, and an energy stability of 0.5%. Throughout the entire experiment, the laser energy is maintained at 1.8 mJ. The femtosecond laser-induced plasma emission spectra are collected and coupled into the fiber-optic probe (core diameter 200 µm) of the spectrometer (Mechelle 5000, Andor, UK) equipped with an intensified charge-coupled device (ICCD) detector (1024 × 1024 pixels, DH334T). This setup utilizes a fused silica lens L2 (f = 75 mm). The spectrometer captures wavelengths in the range of 200 to 975 nm with an accuracy of 0.05 nm and a resolution of λ/Δλ=5000. The ICCD detector has a gate width of 5 µs and a delay time of 4 µs. To prevent excessive ablation of the target samples, Dendrobium samples are mounted on an XYZ-3D translation stage, ensuring that each laser pulse interacts with a new position on the target surface. Additionally, the surface of Dendrobium samples is positioned 1 mm in front of the focal point to minimize interference from air plasma in the spectral analysis. To reduce random errors in spectral detection, 50 laser pulses were accumulated for signal averaging, and 30 LIBS spectral data were collected for each sample. This study primarily conducted LIBS classification analysis on 10 Dendrobium varieties. To ensure the stability and reproducibility of spectral data, the spectra for each Dendrobium variety were acquired from three circular medicinal blocks. Figure 1b displays the LIBS spectra of 10 Dendrobium varieties.

### 2.3. Algorithm Description

#### 2.3.1. Gaussian Filtering and Feature Selection

LIBS technology is a powerful technique capable of simultaneously achieving qualitative and quantitative analysis and providing rich spectral data. However, the complexity of LIBS technology and the high-dimensional nature of its data often result in spectral information containing a significant amount of noise and redundant data, posing significant obstacles to accurately modeling qualitative analysis. Therefore, this study initially performs filtering and preprocessing on the collected Dendrobium LIBS spectral data to achieve preliminary noise reduction. To address the issue of high-frequency noise, Gaussian filtering [39] is employed as a preliminary treatment for high-frequency noise in the LIBS spectral data. Gaussian filtering is based on the Gaussian distribution function, which achieves filtering by applying a weighted average to the signal. The mathematical expression for the Gaussian kernel function, serving as the weighting function for filtering, is as follows:

(1) $$G\left(x\right)=\frac{1}{\sqrt{2\pi \sigma^{2}}}e^{-\frac{x^{2}}{2\sigma^{2}}}$$

where x is the offset from the center, σ is the standard deviation, affecting the width of the curve. Gaussian filtering can effectively suppress high-frequency noise and achieve smoothing processing on the data, making the data more continuous and stable, thereby improving the performance of subsequent algorithms.

Subsequently, we used the maximum mutual information coefficient and the Spearman correlation coefficient to perform feature selection on LIBS spectral data. The maximum mutual information correlation coefficient (MIC) [40] is used to measure the mutual dependence between two random variables. Mutual information $I\left(X;Y\right)$ represents the extent to which the uncertainty of one variable decreases given the value of another variable. The formula for calculating the maximum mutual information correlation coefficient is

(2) $$MIC\left(X,Y\right)=\frac{I\left(X;Y\right)}{\sqrt{H\left(X\right)\cdot H\left(Y\right)}}$$

where $H\left(X\right)$ and $H\left(Y\right)$ are the entropy of variables *X* and *Y*. The Spearman correlation coefficient [41] is a non-parametric statistical method used to measure the degree of association between two variables. It is calculated by first converting the raw data into ranks and then calculating the Pearson correlation coefficient between ranks. Its calculation formula is

(3) $$\rho =1-\frac{6\cdot \sum di^{2}}{n\cdot \left(n^{2}-1\right)}\;$$

where di is the difference for each pair of rankings, and n is the sample size. By first using the maximum mutual information correlation coefficient to select features most correlated with the differences in Dendrobium varieties, and then using the Spearman correlation coefficient to eliminate highly correlated duplicate features selected. Through the above operations, it avoids the interactions between features often overlooked by conventional feature selection methods, truly achieving effective feature selection for LIBS spectral data with high correlation and low redundancy.

#### 2.3.2. Fusion Classification Model

**Figure 2:**
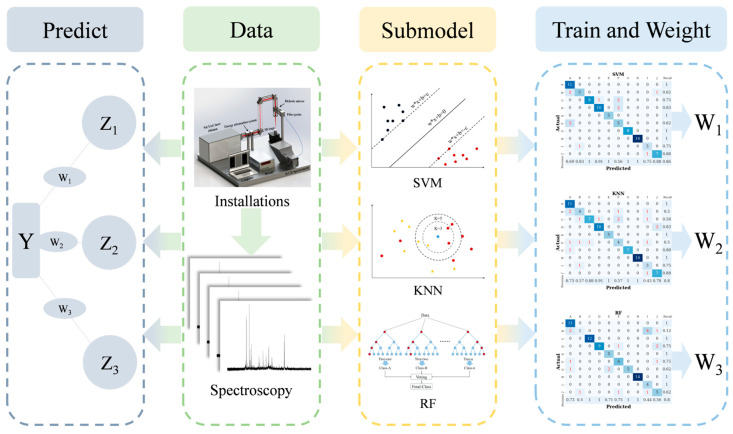
Fusion model structure diagram.

To address the limitations of a single machine learning model in analyzing LIBS spectral data and further enhance the precision and lightweight nature of the model, we propose a fusion classification model for accurate analysis of LIBS spectral data. The overall flowchart of the fusion model is shown in Figure 2.

The fusion model employs SVM, RF, and KNN as sub-models.

SVM [42] is a supervised learning algorithm that, at its core, finds the optimal hyperplane to maximize the distance from support vectors (data points closest to the hyperplane) in the training data. This hyperplane effectively separates data points from different categories, achieving the classification task.

RF [43] is an ensemble learning method that trains multiple decision trees, with each tree trained on a different random subset. Finally, the predictions of all decision trees are combined through voting or averaging to achieve the classification of the target.

KNN [44] is an instance-based learning method that compares a new sample with the neighbors in the training data. This is conducted to find the K-nearest samples for each new sample, and then the category of the new sample is determined by voting. This achieves the classification goal for the target.

Firstly, all sub-models in the fusion model undergo parallel pre-training, and the training precision and recall rates for each Dendrobium species are saved and returned to the fusion model. The training and result-saving process can be represented as:

(4) $$\left(P_{i},R_{i}\right)=G_{i}\left(X\right)\;$$

where $G_{i}$ is the training target mapping function for the *i*th sub-model, and $P_{i}$, $R_{i}$ are the precision and recall returned by each sub-model after training. Then, the classification precision and recall indicators of each sub-model for various Dendrobium species are used as weights. The process of weight setting can be represented as follows:

(5) $$W_{i}=\left(P_{i}*R_{i}\right)$$

where $W_{i}$ is the weight of the *i*th sub-model in the fusion model. Finally, based on the fusion model with added weights, predictions are made for Dendrobium species. The final prediction process of the fusion model can be represented as follows:

(6) $$Y_{i}=\sum_{i=1}^{n}W_{i}Z_{i}$$

where n is the number of sub-models. $Z_{i}$ is the output of the *i*th sub-model. Through the above operations, the different analysis performance of different sub-models on Dendrobium data is combined to achieve a ‘play to strengths and avoid weaknesses’ effect. Parallel training is used for all sub-classifiers during the training process to ensure that the fusion model does not lose its lightweight nature due to the fusion of multiple sub-models.

#### 2.3.3. T-SNE Dimensionality Reduction Visualization

Interpretability has always been one of the most important indicators for researchers and users to measure the excellence of a model. Therefore, to further enhance the interpretability of the entire research process, this study uses t-SNE technology to perform a visual analysis of all the above operations. Using simple and clear two-dimensional images, a more intuitive and comprehensive overview of the global optimization process is achieved, thereby further enhancing the interpretability of the model.

T-SNE is a method used for data dimensionality reduction. It is based on t-distributed neighbor embedding and KL divergence minimization. It achieves non-linear mapping while preserving the local structure of the input data. Compared to linear dimensionality reduction methods, t-SNE visualization technology is more effective in handling non-linear structures. In this study, t-SNE technology is used to reduce LIBS spectral data to two dimensions and display them using visualization tools. Through visualization operations based on t-SNE technology, the entire research process becomes more intuitive and easier to understand. This greatly facilitates the discovery and study of structures and relationships in the data, thereby further demonstrating and enhancing the interpretability of the model.

## 3. Results and Discussion

### 3.1. Control Group Analysis

This study used three widely used machine learning methods (SVM, RF, and KNN) to qualitatively analyze the LIBS spectral data of Dendrobium. Experimental results and observations were recorded for later research analysis and comparison.

Through adding grid search for optimal parameter tuning and applying five-fold cross-validation for evaluation, we obtained the classification accuracy of the three classifiers (SVM, RF, and KNN) for Dendrobium LIBS spectral data as 86%, 80%, and 80%, respectively. The detailed analysis results are shown in Table 1 (accuracy represents the proportion of correctly predicted samples to the total number of samples; recall indicates the proportion of true positive samples correctly predicted as positive; precision represents the proportion of true positive samples among all samples predicted as positive; F1 score is the harmonic mean of precision and recall, used for comprehensive evaluation of model performance). However, these results are still insufficient for practical classification tasks. To further explore the effectiveness of conventional methods in classifying Dendrobium data, we added a common data preprocessing module, Principal Component Analysis (PCA), to the three machine learning methods. After applying PCA for dimensionality reduction on the original data and then inputting it into the classifiers, the experimental results, as summarized in Table 1, revealed that the accuracy of the three classification models with PCA improved to 90%, 90%, and 83%, respectively. In the aforementioned experiment, the average growth rate of each evaluation metric for the three different classification models after adding PCA dimensionality reduction was approximately 5%. However, in some existing literature, SVM, RF, KNN, and other classification models have shown an improvement of 15% or more in each evaluation metric after adding PCA dimensionality reduction, even leading to an overall accuracy close to 100% [45,46,47]. Although the three classification models in this study showed improvement in various metrics after adding PCA dimensionality reduction, they still fall short of achieving precise classification and even fail to reach the cutting-edge level of existing research. The reasons for limiting the classification accuracy of models mentioned above, as analyzed in Section 2.3.1, are that high-dimensional and noisy LIBS spectral data can lead traditional machine learning methods into a “curse of dimensionality” and overfitting problems. Additionally, the mapping rules of a single machine learning model make it challenging to achieve high-precision analysis for such complex data. Even worse, PCA dimensionality reduction techniques often face two main issues when dealing with high-dimensional and noisy LIBS spectral data: firstly, the core objective of PCA is to maximize variance by selecting projection directions, but this does not guarantee the retention of all information, as some valuable information may be distributed in directions with small variances, leading to loss during dimensionality reduction. At the same time, PCA compresses features in a low-dimensional space through linear transformations, making it difficult to fully express the changes in some valuable data, resulting in information loss. Secondly, PCA dimensionality reduction techniques may mistake noisy data for meaningful signals and retain them, causing interference from noise in the reduced data and affecting the analysis results. These factors may lead to results that are not entirely satisfactory.

### 3.2. Data Preprocessing

**Figure 3:**
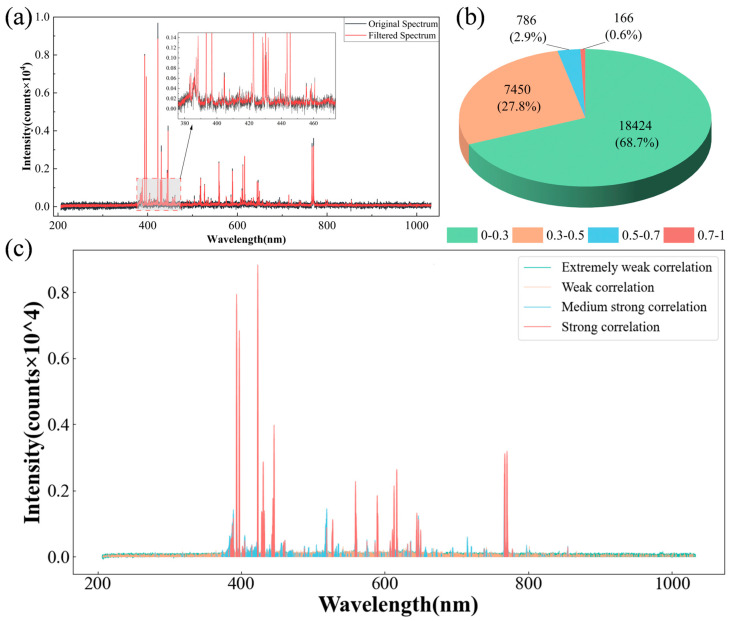
(**a**) Comparison between the original spectrum and the Gaussian filtered spectrum; (**b**,**c**) the proportion and distribution of spectral wave bands with different levels of correlation.

The previous discussion revealed the limitations of PCA dimensionality reduction techniques, making it more challenging to perform reasonable and effective preprocessing on LIBS spectral data. The bottom black spectral line in Figure 3a represents the collected raw LIBS spectrum of Dendrobium. From the spectrum, it is evident that characteristic spectral lines of Dendrobium samples are mainly concentrated in the range of 350–800 nm. Most of the other bands are covered by a significant amount of high-frequency noise, which is a crucial factor limiting the model classification results in Section 3.1. Therefore, we conducted an in-depth investigation into the aforementioned issues with the raw LIBS data and took the following measures to address them. Firstly, we applied filtering to the collected raw LIBS spectral data to reduce the impact of noise on the analysis results. In terms of the choice of filtering method, since the LIBS spectral data collected in this study contains a significant amount of high-frequency noise, Gaussian filtering was selected as the denoising method. Gaussian filtering is a well-recognized method for effectively reducing high-frequency noise. Its basic principle involves using the Gaussian distribution function to perform weighted averaging on the signal, achieving filtering and noise reduction. Regarding the adjustment of the size and standard deviation parameters of the Gaussian kernel required for Gaussian filtering, this study conducted multiple repeated experiments, considering both denoising and preserving effective information. Ultimately, the size and standard deviation of the Gaussian kernel were adjusted to 5 and 1.5, respectively. The top red spectral line in Figure 3a represents the denoised spectrum. It can be observed from the local zoom-in image in Figure 3a that high-frequency noise in the raw data is suppressed, and the trend of the original data is effectively preserved during the denoising process.

Next is the substantial redundant information present in the raw data, considering that the intensity data in most bands of LIBS spectral data contains abundance or concentration information of corresponding elements or molecules, and researchers often highly value the interpretability of the data extraction process. Therefore, we choose the maximum mutual information coefficient, which can retain valid original information and has strong interpretability, to achieve dimensionality reduction. The maximum mutual information is used to measure the degree of mutual dependence between two random variables. In the field of feature selection, it is widely used to assess the correlation between features and the target variable due to its robustness to outliers and noise, low data requirements, and advantages in capturing global feature relationships. By adopting the maximum mutual information coefficient to measure the correlation magnitude between the intensity information of different bands in LIBS and Dendrobium varieties, the specific calculation process is as follows:

(7) $$MIC\left(X_{i},Y\right)=\frac{I\left(Xi;Y\right)}{\sqrt{H\left(Xi\right)\cdot H\left(Y\right)}}\;$$

In the formula, $X_{i}$ represents the data for the *i*th LIBS spectral wave band, and Y represents the Dendrobium variety corresponding to all samples. Then, based on the calculated correlation magnitudes, the most correlated feature data are selected. Figure 3b shows the range of the number of maximum mutual information coefficients for all LIBS wave bands after calculation. It reveals that the number of wave bands with strong, moderately strong, weak, and extremely weak correlation relationships is 166, 786, 7450, and 18,424, respectively. Furthermore, mapping the spectral data of wave bands with different correlation relationships onto the full-spectrum chart, it is clear that in Figure 3c, wave bands with strong and moderately strong correlation relationships indicated by red and blue are concentrated at the peaks of the full spectrum, while wave bands with weak and extremely weak correlation relationships indicated by orange and green are distributed in the remaining lower parts. This is because there are significant differences in the content of various elements in different varieties of Dendrobium. These differences in elemental composition directly lead to differences in the intensity values of corresponding elemental bands in LIBS spectral data. Thus, the results reflected by the maximum mutual information coefficients also confirm the informational content contained in LIBS spectral data. This further demonstrates the rationality and strong interpretability of the aforementioned feature selection results.

The above operation has effectively filtered information for LIBS spectral data, but it only considered the correlation between features and target classes, neglecting the redundancy among features [48]. To consider and further eliminate feature redundancy, we conducted a secondary screening of the already-filtered features. Since secondary screening involves quantifying and judging relationships between all features, an effective and lightweight method is needed to achieve this. However, the calculation of the maximum mutual information coefficient involves non-parametric estimation and requires more computational resources, so it is not suitable for this case. However, the Spearman correlation coefficient has a faster calculation speed and is suitable for nonlinear relationships, making it more suitable for secondary screening. Spearman correlation coefficients were calculated for all features that passed the first screening. The specific calculation process is shown in Equation (3). By setting a threshold for the calculated inter-feature correlation coefficients above, the goal is to eliminate feature redundancy.

**Figure 4:**
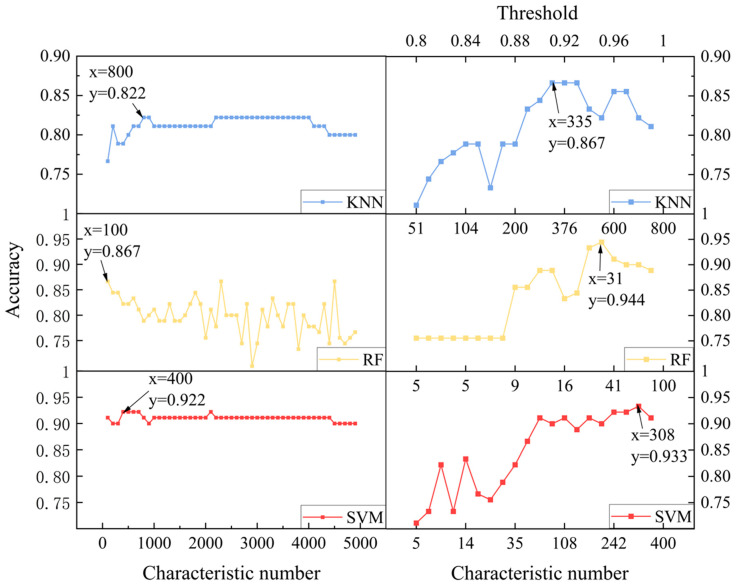
Comparative analysis of the effects of the single correlation coefficient selection method (**left**) and the stacked correlation coefficient selection method (**right**) on SVM, RF, and KNN models.

After this series of experiments and studies, we conducted comparative experiments using the feature data selected by both the single correlation coefficient selection method and the cumulative correlation coefficient selection method as input for SVM, KNN, and RF models to verify the real effectiveness of the operations. First, a grid search was conducted for the single correlation coefficient selection method, with a step size of 50, to search for the optimal parameters in the range of features from 50 to 5000. The grid search results are shown on the left side of Figure 4. From the results, it can be seen that the analysis accuracy of SVM, KNN, and RF models is 0.922, 0.867, and 0.822, respectively, when using the single correlation coefficient selection method as input with feature numbers of 400, 100, and 800. Further, using the feature number corresponding to the highest accuracy of the three models as a benchmark for secondary screening, the cumulative correlation coefficient selection method was used as input. A grid search with a step size of 0.01 was performed to search for the optimal parameter in the threshold range from 0.8 to 0.99. The grid search results are shown on the right side of Figure 4. It is clear that the accuracy of the three classification models reaches 0.933, 0.944, and 0.867, respectively, when the feature numbers are 308, 31, and 335. Compared to the single correlation coefficient selection method, the accuracy has increased by 1%, 7.7%, and 4.5%, respectively. At the same time, the selected feature numbers are only 77%, 31%, and 42% of the single correlation coefficient selection method. Furthermore, when compared to the results of SVM, RF, and KNN with PCA as input, they were 0.90, 0.90, and 0.83. The accuracy of the three models increased by 3.3%, 4.4%, and 3.7% when utilizing the cumulative correlation coefficient method as input. Therefore, whether in terms of analysis accuracy or the degree of data dimensionality reduction, the cumulative correlation coefficient method is significantly superior to the single correlation coefficient selection method and PCA dimensionality reduction method for LIBS spectral data.

When processing LIBS spectral data, the maximum mutual information correlation coefficient not only considers global feature relationships but also takes into account nonlinear relationships in the data. It can effectively filter noise through information theory, efficiently extracting information that truly influences changes in the target class, thereby improving the accuracy and generalization ability of the model’s analysis. Although it effectively extracts features highly correlated with changes in the target class, it ignores inter-feature redundancy. The high correlation presented by inter-feature redundancy can lead to the problem of collinearity. Collinearity makes the model very sensitive to small changes between features, resulting in unstable model coefficients. Using the cumulative Spearman correlation coefficient selection method for secondary screening of the selected features can not only alleviate the collinearity problem but also reduce the complexity of the data to decrease computational costs. Simultaneously, it further reduces the impact of noise on the features of the model. This effectively reduces computational costs and enhances model stability and generalization ability, thereby further improving the accuracy of the model’s analysis.

### 3.3. Fusion Model Construction and Data Analysis

**Figure 5:**
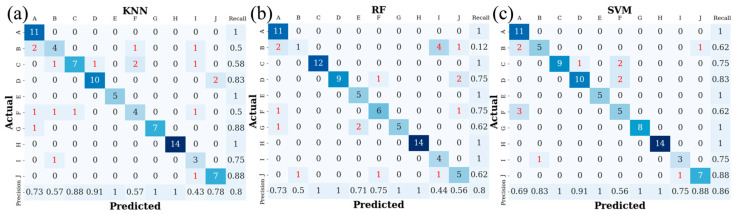
(**a**) Confusion matrix of KNN in control group, (**b**) Confusion matrix of RF in control group, (**c**) Confusion matrix of SVM in control group. The last row of each confusion matrix, cells 1–10, represents the accuracy indicators of different Dendrobium varieties, and the last column of each confusion matrix, cells 1–10, represents the recall indicators of different Dendrobium varieties. The last cell represents the average accuracy of the model.

It is easy to observe from the control group’s experimental results that the mapping rules of a single machine learning model are not ideal for the analysis of LIBS spectral data. However, from the confusion matrices of each model in Figure 5, it can be concluded that SVM achieves 100% classification accuracy for varieties 1, 5, 7, and 8; RF achieves 100% accuracy for varieties 1, 4, 5, and 8; and KNN achieves 87.5% accuracy for variety 2, which is much higher than SVM and RF with 62.5% and 50%, respectively. Therefore, we achieve a complementary fusion of classification models by integrating various models and leveraging their distinct identification performances for different varieties of Dendrobium.

To ensure an objective evaluation of the model’s generalization performance, we divide the input sample data into a validation set, a test set, and a training set in a ratio of 20%, 20%, and 60%, respectively. This method of dataset partitioning effectively avoids overfitting and improves the model’s generalization ability. Additionally, using a test set independent of the training and validation processes for the final evaluation ensures the objectivity of the evaluation results and the authenticity of the model’s performance in real-world applications. During pre-training, each sub-model is trained in parallel with input from the training and validation sets. The classification precision, recall rates, and other evaluation results for each sub-model are recorded and saved. Next, the product of precision and recall rates for each sub-model on different Dendrobium varieties is used as the model’s weight. Through the above steps, the fusion of multiple sub-models is achieved, further improving the classification accuracy with only basic models.

**Figure 6:**
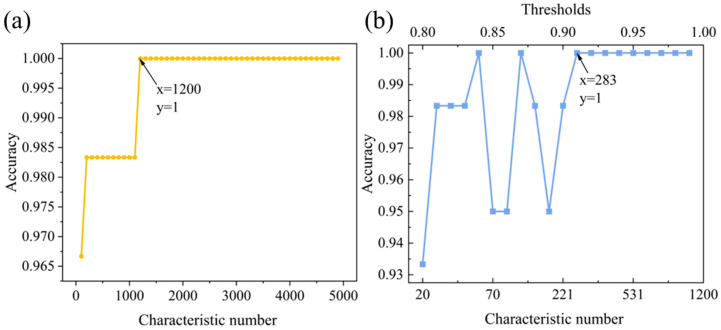
(**a**) shows the model grid search results corresponding to the single correlation coefficient selection method, while (**b**) shows the model grid search results corresponding to the cumulative correlation coefficient selection method.

Finally, we combine the designed preprocessing model with the fusion classification model described above. Firstly, we apply Gaussian filtering to the raw LIBS spectral data for initial smoothing, effectively reducing the impact of noise and laying a preliminary foundation for subsequent analysis. Secondly, we use stacked feature selection to choose the filtered LIBS spectral data, selecting the most relevant features and further removing redundant information between features. This operation not only simplifies the complexity of LIBS spectral data but also effectively reduces the interference of noise in model analysis, enhancing the robustness and generalization ability of subsequent analysis. Finally, by combining the mapping rules of different machine learning models, we utilize the innovative fusion classification model to analyze the results of the above processing, effectively overcoming the limitations of a single machine learning mapping rule in analyzing complex LIBS spectral data. Through the sequential execution of the above three operations in a progressive manner, we step by step enhance the classification accuracy of Dendrobium, achieving precise classification of various Dendrobium varieties. By conducting a grid search to re-optimize the parameters of the overall model, detailed results are shown in Figure 6. Experimental results indicate that the fusion model achieves 100% classification recognition with only 283 features.

**Figure 7:**
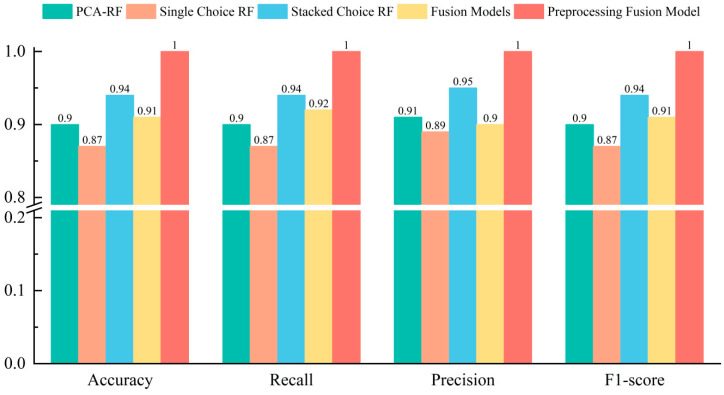
Comparison of multiple model classification results and indicators. To more intuitively verify the effectiveness of the fusion model, only the original LIBS spectral data was used as input for the fusion model. The results are shown in the yellow column, with an accuracy of 0.91. The accuracy of the fusion model has been improved by 0.05, 0.11, and 0.11, respectively, compared to SVM, RF, and KNN, effectively proving the effectiveness and superiority of the model.

In order to verify the robustness of the new Dendrobium species classification method proposed in this paper, it was compared with other models, and the comparison results are shown in Figure 7. It can be clearly seen that the new method proposed in this paper is significantly superior to other models in terms of overall accuracy, precision, recall, and F1 score.

### 3.4. Visual Analysis of the Experimental Process

**Figure 8:**
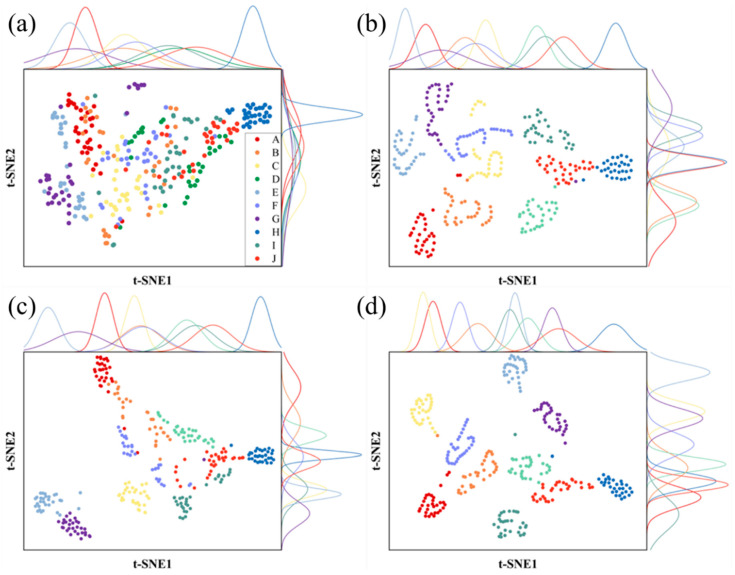
Visualization of t-SNE dimensionality reduction for each operation. (**a**) Original Dendrobium data; (**b**) Data smoothed with Gaussian filtering; (**c**) Data after single-feature selection; (**d**) Data after stacked feature selection. Data points of the same color represent the same Dendrobium variety. The curves on the top and right sides are the normal distribution curves corresponding to the data points of each Dendrobium variety, reflecting the degree of data aggregation.

Finally, to further explain the rationale behind the above operations, this study conducted a t-SNE dimensionality reduction visualization on the data results of the aforementioned operations. The t-SNE dimensionality reduction technique was used to reduce the visualized data to two dimensions and output it to a visualization tool for 2D visualization. Figure 8 shows the t-SNE dimensionality reduction visualizations of Dendrobium raw LIBS spectral data, LIBS spectral data after filtering and smoothing, LIBS spectral data after traditional feature selection, and LIBS spectral data after the proposed feature selection in this study.

In each subgraph shown in Figure 8, each data point represents dimension-reduced Dendrobium LIBS spectral data, and data points of the same color belong to the same Dendrobium variety. The curves on the upper and right sides of the graph are normal distribution curves corresponding to data points for each Dendrobium variety. They intuitively reflect the data’s degree of dispersion. A flat curve indicates higher data dispersion for that variety, while a less flat curve indicates more clustered data, which is more conducive to classification. Additionally, greater separation between curves for different varieties implies better analytical suitability. As seen in Figure 8a, the dimension-reduced data points generated by t-SNE for different varieties of raw LIBS spectral data are dense and intersect. The normal distribution curves on both sides are mostly flat, and there is a high degree of overlap. Therefore, whether in terms of the clustering degree or the dispersion between varieties, raw LIBS spectral data are not ideal. Subsequently, Figure 8b shows that after filtering, the clustering degree of data points corresponding to the same variety is significantly improved. However, there is still some data intersection between certain varieties, and the normal distribution curves on both sides reveal that there is still a relatively high overlap between some varieties. Figure 8c shows that after feature selection with a single correlation coefficient, the intersection of data points between different varieties is effectively reduced, and the clustering degree of data points for the same variety is significantly enhanced. However, the normal distribution curves on both sides reveal that the curves for some varieties are relatively flat, and the degree of dispersion between the same varieties still needs to be further reduced. Finally, Figure 8d shows that after feature selection with the stacked correlation coefficient, data points for the same variety are more clustered, and the normal distribution curves on both sides are generally not flat. Moreover, the curves on the upper side reveal that there is almost no overlap between different varieties. The processed data are ideal and conducive to analysis. From the four images above, it is clear and intuitive that all varieties transition from a state of cross-mixture to becoming more clustered within each variety and dispersed between varieties. This effectively validates the rationality and interpretability of the model operations proposed in this study.

## 4. Conclusions

In this study, we propose a Dendrobium species classification method based on LIBS and machine learning technology, achieving a classification accuracy of 100%. The method involves filtering and preprocessing the raw Dendrobium LIBS spectral data, followed by a weighted fusion classification model to achieve accurate classification. Furthermore, this study utilizes t-SNE dimensionality reduction and visualization techniques to visualize the research process, validating the overall rationality and interpretability of the study. Our classification method shows significant performance improvement compared to traditional machine learning methods such as SVM, RF, and KNN, as well as their improved versions, PCA-SVM, PCA-RF, and PCA-KNN. In terms of accuracy, the proposed classification method in this study achieves a substantial 10% improvement compared to the best-performing PCA-SVM model among the mentioned models. Additionally, there is a qualitative improvement in precision, recall, F1 prediction scores, etc. These performance improvements can be mainly attributed to three aspects. Firstly, this study performed reasonable and effective filtering and denoising on raw LIBS spectral data, reducing noise disturbance while retaining essential data. Secondly, the redundancy in LIBS spectral data was more effectively removed through the feature selection method proposed in this study, enhancing the efficiency of subsequent classification analysis while avoiding the curse of dimensionality. Finally, when constructing the classification model, this study fully considered practical considerations, ensuring its lightweight nature while further enhancing model accuracy. In conclusion, this study has achieved efficient and accurate identification of Dendrobium. Future research could expand the variety of Dendrobium to better meet practical needs. Additionally, this technology could be extended to the classification of other traditional Chinese medicinal herbs, providing a promising new approach and perspective for the identification of other medicinal materials. It offers feasible technical support for the development of the traditional Chinese medicine industry.

## Figures and Tables

**Table 1:** Control group model results.

	KNN	RF	SVM	PCA-KNN	PCA-RF	PCA-SVM
**Accuracy**	0.80	0.80	0.86	0.83	0.90	0.90
**Recall**	0.80	0.82	0.86	0.83	0.90	0.90
**Precision**	0.82	0.77	0.87	0.86	0.91	0.91
**F1**	0.80	0.79	0.86	0.83	0.90	0.90

## Data Availability

The original contributions presented in the study are included in the article, further inquiries can be directed to the corresponding author.
